# The British Journals

**Published:** 1841-01

**Authors:** 


### II. THE BRITISH JOURNALS.

(FOR THE QUARTER ENDING NOVEMBER 30, 1840.)

---

*Case of dangerous Uterine Hemorrhage in which Transfusion was successfully employed, with some observations on the more frequent expediency of that operation.* By RICHARD OLIVER, M.D., Carlisle.

ON the 26th of June 1837, I was called to attend the wife of John Cook, a weaver, living at Eden Place, about a mile from my house. She had been attended by a midwife, and had given birth to a child at its full time in the course of the previous night. The patient was about forty-two years of age, and this was her seventh child. I found her at six A.M. in an exceedingly exhausted condition. Blanched by a profuse hemorrhage, which no adequate means had been employed to suppress, but which had now ceased, she was lying on her back in a state of imperfect consciousness, with the pulse at the wrist barely perceptible, now and then moaning lowly, and casting about her arms. About half a glass of rum with a little water was immediately given to her, and this, with a few spoonfuls of beef tea, was repeated two or three times at intervals of about twenty or twenty-five minutes. After each dose she appeared to be a little refreshed, but upon the whole the symptoms of collapse was gaining ground. About half-past seven o'clock brandy was substituted for the rum, and the dose was increased to an ounce and a half, with the addition of a drachm of aromatic spirit of ammonia and a few drops of tincture of opium to every second or third portion. The same deceitful promises of reaction were succeeded by the same progressive indications of sinking, until at length, about one P.M., she became quite unable to swallow. The pulse at the wrist and in the carotids had not been perceptible for more than two hours and a half, and the coma was now complete. Under these very unpropitious circumstances I determined upon transfusion, with little hope of success, and with no small compunction for having thus afforded the operation so little fair play. At half-past one P.M. I was provided with the apparatus necessary for performing it; and having obtained a willing supply of blood from three of the patient's kind-hearted neighbours, I opened a vein at the bend of her arm, and with the assistance of two of my professional friends, Mr. Bowman, surgeon of this place, and Dr. Henry Lonsdale, now Demonstrator of Anatomy in Queen's College, Edinburgh, I proceeded cautiously and steadily to introduce it.

I had first taken care to see that the instrument was in proper order, and particularly that I should have the syringe and its tubes free from air. After one or two gentle strokes with the piston, made with a view to ascertain this point, I found that the cup attached to the apparatus was so small that it could not be safely used. Unless the piston was elevated very slowly, and the blood was supplied very steadily to the cup, there was great risk of introducing air into the cylinder. But finding that, by taking a common basin to receive the blood, and by drawing it up thence through the bottom of the syringe, I could obviate this danger, I laid the cup aside altogether. With this simpler arrangement I passed syringeful after syringeful into her exhausted veins, pausing from time to time to mark the effects, and anxiously watching for some glimmering promise of the return of energy to her heart. On a moderate computation we had already transfused twelve ounces of blood, and she still lay pulseless and perfectly insensible. The respiration, however, although faint and low, was distinct and regular; so that, however small the amount of blood in her system might be, there was still some undergoing aeration in the lungs; and in gradually augmenting its quantity, we might possibly contribute to raise her vital powers, by enabling a larger portion of it to reach the nervous centres. We could not discern the heart's pulsations, but we might be quite certain that it did beat, and that the general circulation, although thus imperceptible, was still actually carried on. We had yet obtained no assurance of improvement, but it was pretty evident that, by proceeding cautiously, we neither had done nor could do any harm. Without this expedient the poor woman's death was inevitable, and but too probable we then thought even with it; so, disregarding the cautions given upon this point, we determined to go on. Steadily and slowly the blood was introduced as before, until at length we imagined that the pulse became faintly perceptible in the arm; and slight as it was, this intimation of the heart's increased power gave us no small encouragement. After persevering for a few minutes longer, we had the very perfect gratification of witnessing not only the complete restoration of the circulating power, but the return of consciousness, and of the ability to speak. It is unnecessary to advert to the subsequent details of the treatment and of the recovery, farther than to mention that she went on very favorably, and in a few weeks was moving about in her family as usual. She remained for some time longer rather weak and delicate, but beyond an occasional slight headach, and a tendency to constipation and flatulence, she suffered from none of the more prominent and distressing symptoms which ordinarily ensue after serious losses of blood, and she has long been, and still remains, in very good health. With respect to the quantity of blood introduced in this case, I am not able to speak with absolute accuracy; but I feel quite certain that I am below the mark in mentioning twenty-two ounces. From each of the three individuals who supplied the blood, we took at the least an average of twelve ounces; and although we did not attempt to measure the amount of it, I am perfectly satisfied that, at all events, not more than one-third of the whole was lost by coagulation, and by being thrown upon the ground in adjusting and preparing the instrument.

*Edinburgh Medical and Surgical Journal. Oct.* 1840. *No.* 145.

### *Observations on the Diagnosis and Pathology of Fractures of the Neck of the Femur.* By Robert William Smith, A.M., M.R.I.A.

[This is a very valuable Memoir, well deserving attentive perusal. We regret that we can only find room for the tabular view of the cases (each of which is detailed in the paper and illustrated by a woodcut), and the conclusions deduced therefrom by the author.]

| No. | Name. | Age. | Shortening. | Position of the Foot. | Period of Survival after the Receipt of the Injury. |
|---|---|---|---|---|---|
| | *Intracapsular Fractures of the Neck of the Femur.* | | | | |
| 1 | Laurence Maguire . | 40 | ½ inch | Eversion | 14 days |
| 2 | William Collins . . | 36 | ¾ ,, | ,, | 17 ,, |
| 3 | Thomas Maguire . | 84 | ½ ,, | ,, | 14 ,, |
| 4 | Dorah Campbell . | 75 | 1 ,, | ,, | 2 months. |
| 5 | Mary Gill . . . | 80 | ½ ,, | ,, | Not known. |
| 6 | Esther Christie . . | 60 | 1½ ,, | ,, | ,, |
| 7 | Mary Lamb . . | 80 | ¾ ,, | ,, | 1 year. |
| 8 | Margaret Bourke . | 90 | ½ ,, | ,, | 14 days. |
| 9 | Margaret Myler . . | 78 | ¼ ,, | ,, | 2 months. |
| 10 | A Female . . . | 65 | 1 ,, | ,, | Not known. |
| 11 | Patrick Doolan . | 60 | 2 ,, | ,, | 7 years. |
| 12 | Michael Curry . . | 40 | 1¼ ,, | ,, | 1 month. |
| 13 | Matthew Reilly . | 46 | ½ ,, | ,, | 4 months. |
| 14 | A Female . . | 55 | 1 ,, | ,, | Not known. |
| 15 | Sarah Ashton . . | 65 | 1¼ ,, | ,, | 9 years. |
| 16 | Elizabeth Casey . | 50 | ¾ ,, | Inversion | Not known. |
| 17 | Robert Robinson . | 50 | 2 ,, | Eversion | Several years. |
| 18 | Ellen Walker . . | 70 | ½ ,, | ,, | 7 days. |
| 19 | Laurence Reilly . | 56 | 2 ,, | ,, | Several years. |
| 20 | Joseph Seaton . | 90 | 1¼ ,, | ,, | 7 years. |
| 21 | A Female . . | 65 | 2½ ,, | ,, | Several years. |
| 22 | Thomas Connolly . | 50 | ¾ ,, | ,, | 10 days. |
| 23 | Bridget Misset . | 72 | 1 ,, | ,, | 10 weeks. |
| | *Extracapsular Fracture of the Neck of the Femur.* | | | | |
| 24 | Patrick Murphy . . | 80 | 2 inch | Inversion | 14 days. |
| 25 | Alicia Harris . . | 70 | 1½ ,, | Eversion | 5 ,, |
| 26 | James Stanford . | 67 | 2 ,, | ,, | 8 days. |
| 27 | A. B. a man . . | 50 | 2 ,, | ,, | 14 ,, |
| 28 | Mary Kelly . . . | 56 | 1¼ ,, | ,, | 11 ,, |
| 29 | Ellen Bryan . . | 65 | 1½ ,, | ,, | 5 weeks. |
| 30 | Patrick Grant . . | 70 | 1½ ,, | ,, | 5 days. |
| 31 | Margaret Connolly . | 89 | 1½ ,, | ,, | 12 ,, |
| 32 | Thomas Murphy . | 41 | Not known | ,, | A few weeks. |
| | *Impacted Fractures of the Neck of the Femur, external to the Capsule.* | | | | |
| 33 | John Summers . | 74 | 1½ inch | Eversion | 2 months. |
| 34 | Mary M'Kenna . | 52 | ¾ ,, | ,, | 4 days. |
| 35 | Catherine Egan . | 60 | 1¼ ,, | Inversion | 1 month. |
| 36 | Sarah Denny . . | 70 | 1 ,, | Eversion | 1 ,, |
| 37 | Alicia Sherlock . | 64 | ½ ,, | ,, | 15 weeks. |
| 38 | James Power . | 54 | 1½ ,, | ,, | 5 months. |
| 39 | A. B. a female . | 80 | Not known | | Not known. |
| 40 | Not known . . | ... | ... | | ... ... |
| 41 | Bryan Dunn . . | 60 | ¼ ,, | ,, | 13 days. |
| | *Impacted Fracture of the Neck of the Femur, internal to the Capsule.* | | | | |
| 42 | Owen Curran . . | 70 | ½ inch | Eversion. | 1 year & 10 mo. |

From what has been stated in the preceding pages, and from the evidence afforded by the *post-mortem* examination of fifty specimens of fractures of the neck of the femur, forty-two of which have been detailed, I think I am justified in deducing the following conclusions:

1. A slight degree of shortening, removable by the extension of the limb, indicates a fracture within the capsular ligament. 2. The degree of shortening, where the fracture is within the capsular ligament, varies from a quarter of an inch to one inch, or one inch and a half. 3. The degree of shortening, when the fracture is within the capsule, varies chiefly according to the extent of laceration of the fibro-synovial folds which invest the neck of the femur. 4. In some cases of intracapsular fracture the injury is not immediately followed by shortening of the limb. 5. This absence of shortening is generally owing to the integrity of the fibro-synovial folds. 6. In such cases the retraction of the limb may occur suddenly, many weeks after the receipt of the injury. 7. This sudden retraction of the limb, which indicates a fracture within the capsule, is, in general, to be ascribed to the accidental laceration of the fibro-synovial folds. 8. The degree of shortening, when the fracture is external to the capsule and not impacted, varies from one inch or one inch and a half, to two inches or two inches and a half. 9. When a great degree of shortening occurs immediately after the receipt of the injury, we usually find a comminuted fracture external to the capsule. 10. The extracapsular facture is generally accompanied by fracture with displacement of one or both trochanters. 11. The extracapsular *impacted* fracture is generally accompanied by fracture without displacement of one or both trochanters. 12. In such cases the fracture of the processes unites more readily than that of the cervix. 13. The degree of shortening, when the fracture is impacted, varies from a quarter of an inch to one inch and a half. 14. The exuberant growths of bone met with in these cases have been by many erroneously considered to be merely for the purpose of supporting the acetabulum and the neck of the femur. 15. The difficulty of ascertaining crepitus, and of restoring the limb to its natural length are the chief diagnostic signs of the impacted fracture. 16. The position of the foot is as much influenced by the obliquity of the fracture and the relative position of the fragments, as by the action of the muscles. 17. Inversion of the foot may occur in the intracapsular, extracapsular, or impacted fracture of the neck of the femur. 18. When in the intracapsular fracture the lower fragment is placed in front of the upper, the foot is usually inverted. 19. When in the extracapsular fracture with impaction, the superior is driven into the inferior fragment, so as to leave the greater portion of the latter in front of the former, the foot is generally inverted. 20. In cases of comminuted extracapsular fracture without impaction, but with separation and displacement of the trochanters, the foot may be turned either inwards or outwards, and will gene rally remain in whatever position it has been accidentally placed. 21. The consolidation by bone of the intracapsular fracture is most likely to occur, when the fracture is also impacted. 22. Severe contusion of the hip-joint, causing paralysis of the muscles which surround the articulation, is liable to be confounded with fracture of the neck of the femur. 23. The presence of chronic rheumatic arthritis may not only lead us to suppose that a fracture exists when the bone is entire, but also when there is no doubt as to the existence of fracture, may render diagnosis difficult as to the seat of the injury with respect to the capsule. 24. Severe contusion of the hip-joint, previously the seat of chronic rheumatic arthritis, and the impacted fracture of the neck of the femur, are the two cases most liable to be confounded with each other. 25. Each particular symptom of fracture of the neck of the femur, separately considered, must be looked upon as equivocal: the union of all can alone lead to correct diagnosis.

*Dublin Journal of Medical Science. Sept.* 1840.

*On Nervous or Spasmodic Asthma.* By ROBERT J. GRAVES, M.D., of Dublin.

[THE following is only a part of the very interesting section on Asthma, in the article "On the Treatment of various Diseases," in the last number of the Dublin Journal, by this eminent physician.]

It is evident, that to account for the spasmodic symptoms of asthma, we need not have recourse, with Doctor Clutterbuck, to the diaphragm or intercostal muscles, but to the muscles of the trachea and bronchial tubes themselves; on the whole, therefore, we may conclude, that those who have returned to the opinions professed by our predecessors, are not so much mistaken as their opponents pretend. Even when the paroxysm is intense in degree and duration, where the patient is obliged to sit up half the night; where any attempt to lie down produces symptoms of asphyxia; where hours are spent in extreme distress with lividity of face and lips, gasping, loud wheezing, and great fulness of the vessels of the head and neck; even under all these circumstances, the attack may be nothing but a fit of pure spasmodic asthma. A person thus affected may spend a whole night in the way I have described, and yet, towards morning, he may sleep a few hours, and awake refreshed and comparatively free from dyspnœa, and in the course of the day may be able to go up stairs quickly, run, ride, even hunt without difficulty. I have in my recollection, the cases of several young men subject to severe paroxysms of asthma for five or six nights in succession, and who, immediately after the paroxysm disappeared, could use any active exercise as well as the most vigorous and healthy of their companions.

These facts establish the existence of a disease deserving the name of spasmodic asthma, and show that very violent paroxysms of difficult breathing may occur in persons free from organic affection of the heart or lung. When, however, any permanent change in the structure of the respiratory or circulating apparatus exists, then such changes become the exciting causes of paroxysms of dyspnœa, often closely resembling true spasmodic asthma, but readily distinguishable from it, if due attention be paid to the history of the patient's sufferings and his state between the fits. I have now met with so many cases of young persons in whom no trace of any organic complication existed, that it seems to me more than probable, that spasmodic asthma is not so rare a disease as is imagined. In a little boy, some particulars of whose case I formerly published, the attacks were frequent, violent, and to all appearance, purely spasmodic; he got a very severe paroxysm of gout (hereditary from both his father and mother) in his foot, and has never since had asthma, though four years have now elapsed, and he has been subject to all the excitement and violent exercises of a public school. Mr. Fleming, now of the Isle of Man, and Sir Philip Crampton, attended with me a young gentleman, aged about twelve, who was subject to violent dyspnœa, increased by even the most gentle exercise; indeed for many months he could not walk even quietly in his room, without incurring the risk of suffocation for want of breath, attended with palpitation, wheezing, and all the symptoms of approaching asphyxia; every remedy we could devise was tried most perseveringly for a year, without the slightest benefit, when he got typhus fever, from which he narrowly escaped, but since his recovery, he has never had even the least vestige of his former complaint. These two cases exemplify, in a remarkable manner, the influence which the general state of the constitution often exerts on local affections.

Asthma, like all other nervous diseases, is subject to the most unaccountable variations, and is most uncertain as to the effects which our remedies, or the influence of physical agencies, produce. The following is an example. In December, 1839, I attended two gentlemen residing in the same street, and each about forty-five years old; neither was liable to any other disease, and they were both short and stout; on a very cold morning I found one of them very ill indeed; he had not slept at all during the night, and had every moment been no the point of smothering from asthmatic dyspnœa. The extreme violence of the paroxysm he attributed to the fact, that his bed-room chimney had smoked occasionally during the night, and the weather was so cold, that he was afraid to open the window to let out the smoke. I ordered him to change his room, and I then proceeded to visit his neighbour, and found him sitting in a room full of smoke ; he apologized to me for introducing me into so disagreeable an atmosphere, and explained, that when the fit of asthma became very bad, the only sure means of obtaining relief, which he knew of, was to get a good coal fire lighted in the grate, which being done, he made his servant occasionally obstruct the progress of the smoke up the chimney, and thus maintain a certain density of smoke in the room ; this never failed, he assured me, to bring relief. This gentleman was of very active habits, was agent to several large properties, and consequently obliged to travel much about the country ; experience had proved to him, that he could derive no benefit from turf smoke, and therefore he never stopped at any inn where they had no other fuel but turf, as he felt himself insecure unless he could procure coal smoke in case of an asthmatic attack. Such *idiosyncrasies* will ever baffle the researches of the mere morbid anatomists, but afford a useful lesson to the practical physician.

The phenomena of this disease are calculated to throw much light on the nature of what has been termed wheezing. A person subject to asthma, who has been breathing tranquilly the whole evening, may be attacked towards midnight with difficulty of respiration, and a wheezing so loud as to be heard on the stairs; this will continue for several hours, and then terminate, in some with a copious discharge of sputa, *in others without any expectoration whatever.* When we apply the stethoscope to the chest of a person so affected, we hear a great number of bronchitic rales, showing that the larger and smaller tubes are both engorged ; this is a matter of frequent occurrence in cases of dry asthma, where there is no expectoration, and where the fit terminates in a few hours, without leaving behind the slightest trace of pulmonary derangement. Hence we are led to the conclusion, that sounds of various characters and remarkable intensity may be produced without any inflammation whatever, and in fact without any remarkable alteration in the secreting functions of the bronchial mucous membrane, and that these sounds may wholly disappear where there has been no expectoration, and consequently where the bronchial tubes have not been cleared out. This is a fact worthy of being held in memory. Stethoscopists, when they hear bronchial rales, are apt to attribute them to the existence of bronchial inflammation ; but here, with distinct proofs of the absence of inflammation, you may have a *maximum* of bronchial rales, and in the space of a few hours, you may not have a single sound at the very points where so many were audible before. It is obvious, therefore, that some of the received doctrines on the subject of bronchial rales, are still open to discussion. The practical inference, however, to be drawn from this fact is, that we should study such rales with great attention, and in connexion with other signs and symptoms, lest we be induced to treat antiphlogistically a case in which depletion might be uncalled for or injurious, an error by no means unfrequent among those who rely too exclusively on physical signs.

As to the treatment of spasmodic asthma, I have nothing to add to what is generally known, except that it is often serviceable to stupe the whole chest during the fit with flannel wrung out of water, as hot as can be borne ; and that, in some, much advantage is derived from small but very frequently repeated doses of ipecacuanha wine, mixed with an equal portion of good tincture of castor.

*Dublin Journal of Medical Science. Nov.* 1840.

---

### *On the Use of Tincture of the Muriate of Iron in Diabetes Mellitus.*

By CHARLES CLAY, Esq., Surgeon, Manchester.

THE following three cases yielded so decidedly to the use of the tinct. ferr. mur. P. L., after many other remedies had been tried, that I trust that placing them before the public will be the means of testing its merits still further. It is quite necessary, in order to succeed, to give it in large doses, as I have repeatedly tried the same remedy in small doses without any effect. The cases I am about to give were of sufficient standing as to time and obstinacy (and could only be considered bad cases), and of such a character that the trial of any new remedy was perfectly justifiable.

Case i. James Newton, of Ashton-under-Lyne, February, 1836, aged 75 years, had been for two years suffering from diabetic flows of urine, which for nine months had considerably increased; he had been under the care of different persons, and a variety of remedies were tried, but no abatement of the symptoms was observable. When he applied to me the quantity of urine discharged was nine pounds and a half by weight in twenty-four hours, fully charged with saccharine matter; his appearance was emaciated, anxious countenance, and a dry, furred tongue. After trying various plans, without any apparent benefit (with the exception of temporary relief, for a few days, by the exhibition of nitrous acid), at last, without any particular hope of benefit, I ordered the following mixture:

Tincture of opium, ℨjss;

Tincture of muriate of iron, ℨij;

Sulphate of quinine, grs. viij;

Distilled water, ℥ vj. An ounce to be taken three times a day.

After continuing this formula for three days, I was agreeably surprised by a sensible abatement of the quantity of urine, but still as fully charged with saccharine matter; in five days more (that is, eight from the commencement), the abatement continued; the countenance less anxious, tongue clean, and evidently improving in constitution. On the eighteenth day barely four pounds of urine were discharged in twenty-four hours, in which little saccharine matter could be detected. In four weeks, with a continuation of the medicine, he appeared in perfect health, and, at the end of six weeks, ceased taking medicine entirely, and since has had no return of the complaint.

Case ii. W. Grundy, aged 30, of Hurst, came under my care in April, 1838, after being treated by different persons without any apparent benefit. From the decided success of the tinct. fer. mur. in the case of Newton, I began immediately with the same dose, as above stated. The quantity of urine was, at the commencement, eight pounds in twenty-four hours, and full of saccharine matter. For five days no improvement in either the quality or quantity of the discharge was observable; after that time, however, the abatement began to show itself, but without any diminution of the saccharine principle. On the fourteenth day, the diminution of the discharge was remarkable, not more than three pounds and a half in twenty-four hours, and the character of the urine much less sweet. On the twenty-fourth day the discharge was natural in quality as well as quantity, and before the expiration of five weeks he left off taking medicine.

Case iii. Mary Wild, aged 56, of Ashton, had been subject to a diabetic discharge for eight months; her general health had for some time been very precarious, from the cessation of the menstrual discharge: about seven pounds and a half of urine in twenty-four hours. In this case the saccharine matter was not so abundant as in the former cases. I gave the tinct. fer. mur. mixt. for six days, when a slight abatement was observable; but on the twelfth day the quantity was more than at the commencement. On the fifteenth day the abatement again showed itself: and, from this time to the end of four weeks, kept continually decreasing. At this time pleuritic symptoms called for a cessation of these remedies and the substitution of others, during which time a slight increase of urine came on; but on going on with the old medicine the improvement returned. She finally ceased taking medicine at the end of eight weeks, feeling her health quite restored, and has had no return since. The date of this case was March, 1840.

*Lancet. Oct.* 10, 1840.

